# Community-based active-case finding for tuberculosis: navigating a complex minefield

**DOI:** 10.1186/s44263-024-00042-9

**Published:** 2024-02-08

**Authors:** Peter MacPherson, Kwame Shanaube, Mphatso D. Phiri, Hannah M. Rickman, Katherine C. Horton, Helena R. A. Feasey, Elizabeth L. Corbett, Rachael M. Burke, Molebogeng X. Rangaka

**Affiliations:** 1 School of Health and Wellbeing, University of Glasgow, Glasgow, UK; 2 Clinical Research Department, London School of Hygiene & Tropical Medicine, London, UK; 3 ZAMBART, University of Zambia, Lusaka, Zambia; 4 Malawi-Liverpool-Wellcome Programme, Blantyre, Malawi; 5 Department of Clinical Sciences, Liverpool School of Tropical Medicine, Liverpool, UK; 6 Department of Infectious Disease Epidemiology, London School of Hygiene & Tropical Medicine, London, UK; 7 CIDRI-Africa, University of Cape Town, Cape Town, South Africa; 8 MRC Clinical Trials Unit, University College London, London, UK

**Keywords:** Tuberculosis, Screening, Community, Public health, HIV

## Abstract

Community-based active case finding (ACF) for tuberculosis (TB) involves an offer of screening to populations at risk of TB, oftentimes with additional health promotion, community engagement and health service strengthening. Recently updated World Health Organization TB screening guidelines conditionally recommend expanded offer of ACF for communities where the prevalence of undiagnosed pulmonary TB is greater than 0.5% among adults, or with other structural risk factors for TB. Subclinical TB is thought to be a major contributor to TB transmission, and ACF, particularly with chest X-ray screening, could lead to earlier diagnosis. However, the evidence base for the population-level impact of ACF is mixed, with effectiveness likely highly dependent on the screening approach used, the intensity with which ACF is delivered, and the success of community- and health-system participation. With recent changes in TB epidemiology due to the effective scale-up of treatment for HIV in Africa, the impacts of the COVID-19 pandemic, and the importance of subclinical TB, researchers and public health practitioners planning to implement ACF programmes must carefully and repeatedly consider the potential population and individual benefits and harms from these programmes. Here we synthesise evidence and experience from implementing ACF programmes to provide practical guidance, focusing on the selection of populations, screening algorithms, selecting outcomes, and monitoring and evaluation. With careful planning and substantial investment, community-based ACF for TB can be an impactful approach to accelerating progress towards elimination of TB in high-burden countries. However, ACF cannot and should not be a substitute for equitable access to responsive, affordable, accessible primary care services for all.

## The shifting landscape of tuberculosis epidemiology and its relevance for community-based active case-finding programmes

Tuberculosis (TB) killed approximately 1.6 million people in 2021, second only to COVID-19 among infectious diseases [1]. Recent progress towards global targets to end TB as a public health problem has been mixed at best and severely lacking for many regions of the world [1]. In Africa, for example, TB incidence and mortality both declined substantially between 2000 and 2020, likely mainly driven by the rapid scale-up of high population coverage of effective treatment for HIV [2]. However, progress in other regions—notably in the Americas, South-East Asia, and the Eastern Mediterranean—has been considerably slower [1]. Global TB elimination targets are very unlikely to be met by 2035, and prospects have been worsened by the COVID-19 pandemic [1].

In addition to directly causing widespread illness and mortality in high TB burden countries, the COVID-19 pandemic severely disrupted healthcare systems [1]. This resulted in interruptions of TB screening, diagnosis, and prevention programmes due to closure of health facilities; redirection of medical and public health resources from primary and secondary care to the COVID-19 response; fear among health workers and stigmatisation of people with cough due to the similarity between TB and COVID-19 symptoms, resulting in longer diagnostic delay; and high levels of sickness and mortality among frontline health workers [3]. An estimated 1.3 million people who developed TB had their diagnosis and treatment delayed or missed during the emergency response to COVID-19 in 2020 [4, 5]. This has likely contributed to overall increases in transmission of TB (particularly after initial “lockdowns” had ended), with estimated incidence increasing by 4.1%, from 10.1 million people in 2021 to 10.6 million people in 2022, reversing previous slow downward trends in global TB incidence [1]. The substantial investment and expansion in rapid nucleic acid amplification testing (NAAT) platforms for COVID-19 contrast starkly with years of under-investment in TB diagnosis. The longer-term after-effects of the COVID-19 pandemic on efforts to eliminate TB are uncertain, but this pandemic has undoubtedly made an uphill battle even steeper.

In many countries, TB is predominately a disease of urban dwellers, with high rates of transmission driven by crowding, exposure to poor air quality, and poor access to health services [6, 7]. National TB prevalence surveys—particularly in Africa—show that people who live in cities often have a higher prevalence of undiagnosed TB compared to people living in rural areas [8, 9]. As TB incidence falls in many African countries, there is early evidence to suggest emerging hyper-concentration of people with undiagnosed TB *within* cities and in peri-urban settings [10, 11]. Statistical modelling of data from citywide prevalence surveys linked to spatially-resolved notifications in Malawi and Uganda indicates a high degree of heterogeneity, with informal urban and peri-urban neighbourhoods—often home to recent migrants to the city—being hotspots [10, 11]. In Peru, high rates of TB transmission inferred from genomic analysis have indicated high rates of transmission of multidrug-resistant TB concentrated within neighbourhoods with strong epidemiological links to prisons, with the prison-informal settlement nexus acting as a “transmission amplifier” [12].

These shifts in TB epidemiology are to be expected; public health practitioners have long recognised that TB concentrates in the most deprived neighbourhoods where precarious living conditions facilitate transmission and people are susceptible to disease progression and frequently are not given the opportunity to access timely medical care [7]. Beyond neighbourhoods, intersecting risk factors for the development of TB include older age, male sex, immunosuppression including through HIV infection (partially, but not fully, mitigated by antiretroviral therapy), diabetes mellitus, under-nutrition; and alcohol use, tobacco use, and injecting drug use [13]. Where infection control procedures are suboptimal, busy healthcare centres and other congregate settings within cities (such as prisons, or centres for people experiencing homelessness) can amplify transmission of both drug-sensitive and drug-resistant TB [14, 15]. Recent analysis of data from community TB prevalence surveys has highlighted the importance of subclinical TB as an important contributor to transmission [16, 17]. People with microbiologically positive sputum results, but who either do not have, do not report, or have fluctuating TB symptoms, can transmit TB to others. Earlier diagnosis of subclinical TB, for example by chest X-ray screening followed by microbiological testing of sputum, could then diagnose TB earlier, reducing the infectious period.

Beyond the continuing high incidence and mortality rates from TB, it is imperative to keep in clear focus the people at the heart of this pandemic. Individuals affected by TB frequently experience prolonged periods of illness prior to diagnosis [18], and even if diagnosed and successfully treated, often have substantial limitations in function and wellbeing due to post-TB lung disease [19]. For households, the consequences of TB can be catastrophic, with young children exposed to TB particularly susceptible to rapidly progressive severe disease with high mortality [20], and loss of household livelihoods accelerating cycles of poverty and ill health [21].

Given the continued unacceptably high rates of undiagnosed TB in many communities around the globe, there has been a renewed interest in community-based active case-finding (ACF) programmes to find, diagnose, and treat the “missing millions” of people with TB. ACF programmes are perhaps one of the longest-running screening programmes delivered globally [22]. However, the evidence base for their effectiveness is sparse, and there have been few attempts to synthesise available data and practical experience to guide impactful implementation that achieves maximum public health benefit, whilst respecting the rights of individuals and minimising their exposure to harm [23]. ACF programmes are often major undertakings, requiring substantial funding and demands upon health systems and communities. Yet, despite being implemented with good intentions, they are often delivered in a way unlikely to achieve meaningful and lasting public health or individual benefit. As we enter a new era in the global and regional epidemiology of TB—and in our public health, medical, social, and political response—new approaches to delivering ACF are required.

Here, we argue that ACF programmes need to be designed, delivered, and evaluated with greater precision and with thoughtful, context-specific consideration. We believe that there is a huge opportunity to reinvigorate ACF programmes not only to catalyse reductions in incidence and mortality from TB but also to address the huge burden of human suffering caused by the TB pandemic in a sustainable and respectful way that recognises and attempts to minimise potential harms from screening. We propose using high-resolution epidemiological data to target ACF programmes to communities where they will have the greatest likely benefit, based on local public health needs assessment, and supported by ongoing monitoring and evaluation against meaningful metrics. We recognise that the emergence of several new TB screening and diagnostic tools offers a great opportunity to speed up TB case detection and address the issue of subclinical TB, but their deployment is often limited by cost and practical considerations; we will discuss how the use of these tests can be optimised to provide more efficient and accurate screening algorithms, whilst ensuring affordability for programmes and minimising false positive diagnoses. Finally, we consider how community-based ACF programmes can provide greater support to individuals, households, and communities as part of integrated public health programmes, offering bilateral screening for co-morbidities, and addressing the underlying determinants of ill health, providing maximum return on investment for Ministries of Health. Importantly, benefits from TB ACF programmes may only be fully appreciated in population health over decades, and so a long-term planning horizon is required.

## What is community-based active case finding and evidence for effectiveness

Community-based ACF has been defined by WHO as systematic screening for TB outside health facilities among populations at risk [23]. ACF, which implies a direct contact between a community health worker and participant where screening for TB is offered, differs from enhanced case finding (ECF), where advocacy, communication, and social mobilization activities are intended to prompt earlier care-seeking for TB symptoms at healthcare facilities [24]. The rationale behind ACF is that, by offering screening to people at risk of TB, they will receive an earlier diagnosis and treatment initiation than they otherwise would have done had ACF not been available. This will potentially reduce individual morbidity and mortality whilst also reducing the number of individuals with prevalent infectious TB in the community and shorten the duration of infectiousness, leading to reduced TB transmission, and which will ultimately result in accelerated reductions in TB incidence over time. ACF programmes are frequently accompanied by additional TB-focused programmes, such as health promotion messaging, intensified case finding within health facilities, laboratory strengthening, introduction of new diagnostic tests, and expanded testing and treatment for latent TB infection [22]. This means that the impacts of community-based ACF may be difficult to isolate. We also note that, due to suboptimal detection in nearly all countries, and the complex and long natural history of the disease, the incidence of TB cannot be directly measured, and so proxy measures of the epidemiological impact of ACF programmes must be used (such as case notification rates or cross-sectional TB prevalence surveys) [25].

In the 2021 Guidelines for Systematic Screening for Tuberculosis Disease, WHO recommended that systematic screening for TB through ACF and other interventions could be offered to general populations where the estimated prevalence of undiagnosed TB is above 0.5% (500 per 100,000) adults [26]. This is an expansion of the previous 2013 guidelines, where a threshold of 1% (1000 per 100,000) was recommended [27]. The updated 2021 recommendation was conditional, based on low certainty of evidence. Given that community-based ACF programmes have been implemented since the 1940s (at least 80 years ago) [28], that evidence remains uncertain emphasises the challenges in conducting high-quality evaluations.

In the 2021 WHO guidelines, evidence was synthesised to evaluate the effectiveness of community-based ACF programmes against population- and individual-level outcomes [26]. At the population level, nine studies (two of which were community cluster-randomised trials) investigated the impact of ACF programmes against the prevalence of TB (i.e. the percentage of the adult population with microbiologically confirmed pulmonary TB), with a wide variety of implementation strategies and screening algorithms that precluded meta-analysis [22]. The non-randomised studies had substantial methodological limitations, meaning that the effects of ACF were challenging to interpret [22].

Of the two cluster-randomised trials considered in the WHO Guideline Development Group meeting, the ZAMSTAR Study in Zambia and South Africa—which included a combination of ECF activities and sputum drop-off points for microscopy—did not affect TB prevalence [29]. In contrast, the ACT3 Study in Vietnam, a more intensive intervention comprising 3 years of repeated rounds of TB screening with sputum Xpert testing for all able to produce a sample, resulted in a 44% relative reduction in the prevalence of microbiologically confirmed TB [30]. Both ZAMBART and ACT3 conducted testing for *Mycobacterium tuberculosis* (Mtb) immunoreactivity in sentinel schoolchildren populations to evaluate the effectiveness of transmission. In an analysis of pre-specified outcomes, neither study demonstrated the effectiveness of ACF on Mtb immunoreactivity, although in the ACT3 study a post hoc analysis of interferon gamma release assay (IGRA) results among children who would have been 3–10 years old when the intervention started showed a 50% relative reduction in children from communities exposed to the ACF programme.

Since the publication of the 2021 WHO TB screening guidelines, two further community cluster randomised trials that set out to evaluate the impact of ACF on TB prevalence have reported results. The TREATS Study (also in Zambia and South Africa) offered 4 years of repeated rounds of door-to-door systematic symptom screening for TB and chest X-ray with analysis by computer-aided diagnosis (CAD) software, followed by Xpert testing (HIV-positive) or smear microscopy (HIV-negative) for those symptomatic or with a high CAD score (≥ 50) [31]. Overall, there was no significant difference in TB disease prevalence [31] or Mtb immunoreactivity [32] between intervention and comparison communities. The SCALE Study, done in Blantyre, Malawi, set out to investigate the effect on TB prevalence of door-to-door symptom enquiry for TB, followed by sputum microscopy for those with cough ≥ 2 weeks [33]. Analysis of pilot data showed that the prevalence of TB disease in Malawi had substantially declined, meaning the study was underpowered to detect the expected impact. This, combined with the disruptions due to the COVID-19 pandemic meant that the trial primary outcome was redefined to be a comparison of case notification rates, rather than prevalence. Analysis of the redefined primary outcome showed no increase in tuberculosis notifications from the previously successful approach targeting symptomatic disease, likely due to previous TB ACF programmes and rapid declines in TB burden.

A larger number (*n* = 28) of randomised and non-randomised studies have evaluated the effect of community-based ACF on case notification rates (i.e. the number of people registered as starting TB treatment per 100,000 population) [22]. Nearly all studies showed that, where ACF was implemented, case notification rates increased contemporaneously and to a greater extent than in comparison areas. These findings are supported by published [34] and unpublished data from TB REACH implementation projects, funded through the StopTB Partnership since 2010 to evaluate innovative community-based ACF programmes in high TB burden countries, which often led to substantial increases in notifications [35]. However, there were limitations in the design and evaluation of most of these studies. Non-randomised studies are subject to selection bias, where communities with the highest burden of undiagnosed TB are likely to be preferentially chosen to receive ACF programmes [22, 36]. TB REACH projects are also preferentially focused on marginalised communities with poor routine health access [34]. Moreover, funding was of short duration and nearly all studies only investigated trends in case notification rates *during* the implementation of the programme (and occasionally before) [22]; to the best of our knowledge, only two studies (one done in Brazil [37] and one in Malawi [38]) compared trends in the post-ACF period. This is important as historical evidence and modelling show us that the effects of once-off ACF on underlying TB epidemiology and diagnosis are only likely to be temporary, with repeated rounds and multi-year follow-up required for lasting effect on underlying population epidemiology [39, 40]. First-round effects may differ from subsequent rounds due to meeting “pent-up” demand for services in the initial round, novelty value, or alternatively increasing effort and experience of the programme team if initial results were disappointing. Analysis from Miller et al. [37], and a more recent evaluation of re-initiating ACF in a community with previous ACF programmes [41], has shown that a single round of ACF does not necessarily lead to sustained differences in case notification rates between intervention and comparison communities. Widespread publicity and knowledge of the ACF programme itself probably increase routine diagnosis (i.e. out with the ACF programme) in both ACF and—to a lesser extent—comparison communities [41]. This indirect “health promotion effect” could be an important, but under-appreciated, contributor to the overall impact of ACF programmes. Finally, often studies report ACF programmes that target areas that are especially poorly served by existing primary care, and thus the ACF impact could represent the impact of having a (temporary) service to meet health seeking demand for TB diagnosis that could and should be met through equitable access to responsive and well led primary care service. In contrast, in some very high TB burden settings, ACF maybe needed in addition to even-high quality primary care in order to end TB epidemics in a timely fashion.

Further systematic reviews for the 2021 guidelines investigated the individual-level impact of ACF [42] and the impact of ACF on knowledge, attitudes, and care-seeking behaviours [41]. However, in each case, there was very little evidence identified. For individuals participating in ACF programmes, potential benefits include earlier diagnosis of TB, which could reduce the length of sickness and risk of death and potentially impact the severity of post-TB sequalae, ultimately improving quality of life. Many people with symptoms of TB delay care-seeking due to pressure of time, competing household and work demands, and lack of accessible primary health care services; community-based ACF programmes could bring services closer to communities and potentially reduce care-seeking costs and the catastrophic household costs associated with TB diagnosis and treatment. There are also opportunities to increase knowledge and awareness of TB among individuals in high TB burden communities, potentially prompting earlier care-seeking. Finally, where other health programmes are combined with ACF, individuals may receive additional screening services or programmes such as access to HIV testing and treatment.

In contrast, there are potential harms to individuals from participating in ACF programmes. These include the risk of false positive TB diagnosis, necessitating unnecessary (and potentially harmful) TB treatment; as TB prevalence declines screening and diagnostic algorithms should be carefully designed to minimise false-positive results. People identified with TB may experience stigmatisation or discrimination from communities, especially where ACF programmes and individual-level follow-ups are delivered close to households; care should be taken to ensure privacy and confidentiality. Where testing for Mtb immunoreactivity is offered, the benefits to some individuals from TB preventive therapy are not clear (e.g. guidelines recommend use for young children and people living with HIV, who are at increased risk of development of TB disease, but there is not strong evidence to support use for other groups). Research is required to better understand the potential tensions between population and individual harms and benefits from ACF, and this should be embedded within future programmes.

Taking all of this together, it is clear that the evidence base supporting community-based ACF programmes is mixed. It is probable that repeated rounds of high-intensity ACF offered to communities with a high burden of undiagnosed TB and where the population and health service are highly motivated to support ACF programmes can rapidly reduce prevalence to an extent likely to have an immediate impact on TB, but less intensive programmes are at high risk of having negligible population-level impact despite high resource requirements. ACF also very likely increases TB case notifications, at least for the first few rounds of ACF, but the sustained impact beyond the ACF period or of repeated rounds is mostly unknown. There are critical knowledge gaps about what the benefits and harms are for individuals participating in ACF programmes and how these can change community and individual knowledge and behaviour around TB. Overall, most community-based ACF studies reviewed do not clearly define the population intended to benefit from the ACF programme, nor provide a plausible logic model for how sustainable impact will be achieved or rigorously evaluated. Although WHO now conditionally recommends a threshold of above 0.5% prevalence for offering community-based ACF to general populations, national TB prevalence surveys do not provide the precision to allow identification of priority populations for ACF, either at a city level, or *within* cities, or indeed by intersecting population characteristics such as age and sex. Given the resources and community engagement required to undertake evaluations of ACF programmes, we strongly recommend that researchers and health programmes considering implementing a community-based ACF programme carefully consider how to optimise delivery to maximise initial impact, maintain engagement and participation in subsequent rounds, and embed rigorous evaluations within their programmes to provide much-needed evidence. We provide some practical approaches to do this below.

## Who stands to benefit from community-based active case finding?

When considering funding, planning, implementing, and evaluating a community-based TB ACF programme, researchers, policymakers, and planners should take a public health approach to consider who stands to benefit, by how much, and at what costs (Table 1). Populations should be prioritised where disease burden is highest and where benefits are likely to be greatest, maximising efficiency in the use of limited health-sector resources. As TB is an infectious disease, targeting ACF towards priority groups with undiagnosed infectious disease will likely have a disproportionately greater and more rapid impact on transmission than programmes that are untargeted or targeted towards population groups responsible for a smaller overall fraction of transmission events [43]. Once sustained reductions in disease burden in priority groups have been achieved, then ACF programmes can possibly be expanded to other communities and groups who may receive individual-level benefits or contribute further—though likely smaller—impacts on transmission.

**Table 1:** Potential benefits and harms to populations, health services, and individuals from TB active case finding programmes

	**Potential benefits**	**Potential harms**
**Populations and health services**	• Reduced TB transmission • Reduced TB incidence • Improved TB case detection • Reduced TB mortality • Integration of other health, surveillance, and development programmes	• Repeated rounds of intrusive and inconvenient programmes • Potentially draw resources from other health priorities and programmes if not carefully designed and implemented • Overburdened/overstretched health workforce • Potential loss of focus on primary healthcare strengthening and universal healthcare access due to “vertical approach”
**Individuals and households**	• Earlier TB diagnosis and treatment • Reduced sickness and debility from TB and post-TB sequalae • Potentially reduced care seeking/household catastrophic costs • Opportunity to increase knowledge/awareness/behaviours around TB • Receipt of other health programmes co-delivered with ACF (e.g. HIV, viral hepatitis, leprosy, non-communicable diseases) • Improved quality of life	• False positive TB diagnosis, necessitating unnecessary TB treatment • Potential to increase stigmatisation within communities and neighbourhoods • Uncertain benefit of TB prevention programmes for some population groups (e.g. TB preventive therapy)

Based on the established and recently changing epidemiology of TB, we recommend that most high-TB burden countries, cities, and particularly high-density informal settlements within cities should be the highest priority, although in some countries (e.g. Brazil, China, for example, which have greater epidemics in rural populations) selection of target populations should be guided by good understanding of local epidemiology at national and subnational level. We stress that decisions about where to implement ACF programmes are primarily a resource-allocation issue, recognising that inefficient deployment will likely draw resources away from other priority populations affected by TB and other health and development programmes. For a country with a predominately urban-concentrated epidemic, it is unlikely that ACF programmes primarily focused on rural areas will achieve a substantial yield of diagnosis to appreciably alter transmission dynamics; of course, there are likely to be exceptions, and the decision to fund ACF programmes should be fully justified by a detailed impact assessment, cost-benefit analysis, and evaluation plan.

**Fig. 1:**
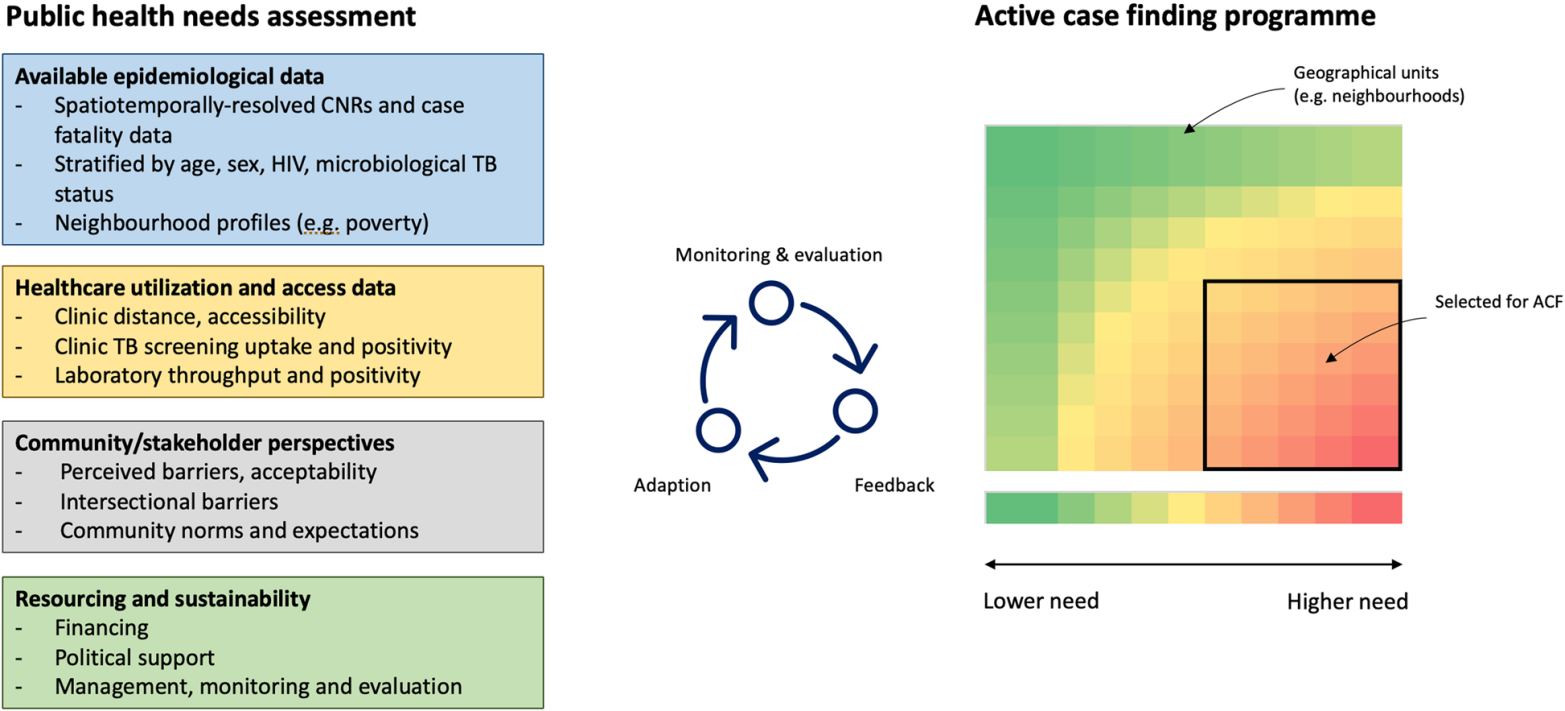
Public health needs assessment for planning, monitoring, and adapting community-based active case-finding programmes. ACF active case finding, CNR case notification rates

Knowledge of where ACF programmes should be targeted is then dependent upon the detailed local understanding of epidemiology, geography, demographics, and health service access and utilisation; this requires close partnership with affected communities, leaders, and decision-makers, supported by careful appraisal of all available data and planning. The public health approach to making such an assessment is known as a needs assessment [44] (Fig. 1), and we—like WHO—argue that no ACF programme should be conducted without this being done.

Two systematic reviews have synthesised data from studies that have attempted to target ACF programmes towards priority populations or geographical areas (“spatial targeting”), finding that the very limited evidence available shows that both design and evaluation of spatially targeted interventions are usually done on an ad hoc basis, without careful consideration of these points [45, 46]. In the absence of data on local TB prevalence, other epidemiological data can be triangulated—perhaps supplemented with rapid community surveys [47]—to provide insights about where ACF should be first deployed. This could include health indicator data on the distribution of poverty indicators within cities (e.g. through census data, household surveys, high-resolution spatial datasets [48], or participatory wealth ranking methods [49]); trends in TB case notification rates and case fatality ratios disaggregated to the neighbourhood level or geolocated using novel mapping tools (although with the caveat that low case notifications may represent poor access to care rather than a low burden of disease); HIV care indicators, including spatially resolved rates of diagnosis of advanced HIV disease; and high-quality qualitative interviews with key stakeholders. We note that targeting ACF towards priority populations in cities is not a new concept; indeed, in mass chest X-ray screening programmes in the USA and Europe in the 1940s–1960s explicitly prioritised poorer neighbourhoods within cities for screening [28, 50]. Within hotspot areas, high coverage with sensitive tests across the population can be achieved by otherwise untargeted delivery, as with the ACT3 trial that has provided the strongest evidence for transmission reduction from active case finding [30]. However, even within “hotspot areas” particular groups—typically men and older people—will have substantially higher prevalence of undiagnosed TB than others. If compromises have to be made, then greater efficiency and effectiveness may then be achieved by prioritising these groups for active case finding, especially if these same high-risk sub-groups also have other “hard to reach” characteristics leading to below average participation rates, although we have no date to support this hypothesis. In this context, it is also clear that more highly targeted screening strategies will miss a high proportion of undiagnosed TB patients, as will interventions that rely on self-identification of symptoms.

Targeting ACF to population groups in congregate settings may also be an effective and efficient way to rapidly reduce TB transmission. Examples include ACF programmes offered to people deprived of their liberty in prisons [51] (which may also cause “spillover” into the community [52]), people who work in mines [53], and interventions explicitly targeted towards men [54], for example at transport hubs [55], bars, or through sporting events. Where populations are ageing—such as in many Asian and Western Pacific region countries—careful consideration of targeting ACF programmes towards older populations will be required [56].

Programme design and evaluation strategies should be embedded within the selection of target populations, with key considerations including: careful design of community-engagement and publicity campaigns; selection of a screening and confirmatory testing algorithm; timing of rounds of ACF, and overall length of programme likely to achieve maximal impact; establishment of patient-centred pathways for TB treatment and prevention services; availability of a clinical evaluation and management service for participants identified with non-TB diagnoses as part of the screening, which are likely to considerably outnumber TB diagnoses [57]; laboratory strengthening, and quality assurance systems; data management and security; and development of a rigorous monitoring and evaluation plan that will allow assessment of the epidemiological and individual participant impact of the ACF programme, appraisal of cost-benefit analysis, and contribute to current knowledge and evidence gaps. Because of the size and scale of ACF programmes, it can be highly efficient to nest sub-studies within them, for example, evaluation of the diagnostic accuracy of novel screening tools.

Screening and diagnostic testing algorithms will necessarily be guided by available resourcing. Screening algorithms can include an initial symptom screen (with different symptom combinations providing trade-offs in terms of sensitivity and specificity for microbiologically-confirmed pulmonary TB) [26, 58], followed by a confirmatory test: sputum smear is relatively cheap, and in well-functioning systems can achieve moderate sensitivity [59], whereas molecular testing with NAAT provides gains in sensitivity—particularly for people living with HIV [60]—at substantially greater cost. Strategies such as the universal offer of sputum nucleic acid amplification (NAAT) testing regardless of symptoms (as in the ACT3 Study [30]) are likely to be more effective but with substantially greater programmatic costs [61].

An alternative strategy is to offer chest X-ray screening, which can detect subclinical TB (i.e. people who have microbiologically-confirmed TB in sputum but do not report symptoms), with interpretation either by human readers (e.g. radiologists, radiographers, or other trained health workers), or by CAD software [26, 62–64]. Chest X-ray is affordable and fast, highly acceptable, and can be deployed in a wide variety of settings with the recent development of mobile and ultra-portable systems [65]. As an estimated 50% of people with prevalent TB in communities are estimated to have subclinical TB [16] and are thought to make a substantial contribution to TB transmission [66], this suggests that chest X-ray screening could provide additional population benefits. Indeed, the mass X-ray campaigns in Europe and the USA in the mid-twentieth century explicitly recognised that, from a public health perspective, identifying TB early in its asymptomatic or pauci-symptomatic stages was a central benefit of this approach [28, 50]. However, evidence from some settings, such as Blantyre, Malawi, which have seen rapid declines in TB prevalence in the absence of widespread use of chest X-rays, suggests that the epidemiological importance of subclinical TB may be overstated [67]. High-quality evidence evaluating the impact of screening for subclinical TB on population- and individual-level outcomes is sorely needed. Moreover, the potential population benefits of wider X-ray availability in primary care for TB programme and more broadly are mostly unknown.

There may also be potential to further improve the efficiency of chest X-ray screening, as confirmatory sputum testing still forms the greatest fraction of programme costs. With CAD software, operators can select a “TB abnormality” threshold, above which sputum testing would be recommended. However, individual-level characteristics appear to have a large effect on CAD specificity, with older age, previous TB history, and possibly HIV infection increasing false-positive screens [68, 69]. Setting adaptive thresholds based on individual characteristics could dramatically decrease the number of confirmatory sputum tests required, without impacting sensitivity; prospective studies and modelling are required. An alternative approach could be to introduce a second screening test (perhaps C-reactive protein) to follow a chest X-ray where the CAD score falls between a moderate range of values; this could reduce the number of sputum tests required and increase the prior probability of TB among those undergoing confirmatory sputum testing, whilst maintaining overall sensitivity and specificity. Again, such approaches require validation in carefully designed studies.

There have been repeated calls to integrate community-based ACF programmes with other health services for several years (e.g. [70]), including from ourselves over a decade ago [71]. Here, we go further and argue that it is a moral imperative to ensure that limited health system resources are effectively and efficiently used, even if this requires concerted preparatory work and collaboration across traditional disease “silos”. Integrating HIV testing, treatment, and prevention services within ACF programmes is probably the most common approach developed, and several large randomised trials have included innovative approaches to the deployment of new HIV self-testing and home treatment initiation services [72, 73]. In PopART in Zambia and South Africa, community health workers delivered a rigorously evaluated HIV-prevention intervention with ACF screening [31]. As the prevalence of undiagnosed HIV and TB often share geographical distributions and risk profiles, combining ACF with an offer of new long-acting injectable HIV pre-exposure prophylaxis within community-based ACF programmes could be impactful in addressing both TB and HIV incidence [74]. In some settings—but not all—interventions to screen for non-communicable diseases such as diabetes mellitus and hypertension may be possible, although the individual and population-level impacts of such programmes are unknown. Given that ACF interventions are often delivered over multiple rounds through door-to-door interventions and sometimes with community mobile clinics where biological samples (sputum, finger-prick blood samples for HIV testing) are already taken, there is an opportunity to integrate public health surveillance programmes for other infectious diseases [75]. Examples could include blood-based serosurveys for arbovirus infections or COVID-19, nasopharyngeal sampling for viral respiratory infections, or syndromic surveillance for emerging infectious diseases [75]. As always, decisions should be guided by local epidemiology and a thorough needs assessment with the needs of communities and community input at the centre of decision-making.

## Evaluation of the impact of ACF programmes

In the absence of a test of recent infection for TB that could allow inference to be made about true incidence, it remains challenging to select outcomes to evaluate the public health impact of community-based ACF programmes. Measurement of the prevalence of microbiologically confirmed pulmonary TB in a random sample of adults selected from intervention and comparison communities probably remains the gold standard outcome, but is logistically challenging to undertake, requires excellent quality-assured TB laboratories providing NAAT testing, smear and culture (either as part of the microbiological case definition, or for quality assessment), and is extremely expensive [22]. Given these barriers, only three published randomised trials and one before-after comparison (to the best of our knowledge) have evaluated ACF effectiveness against a prevalence outcome, and all have struggled to achieve high levels of sputum collection and results from participants [22].

**Fig. 2:**
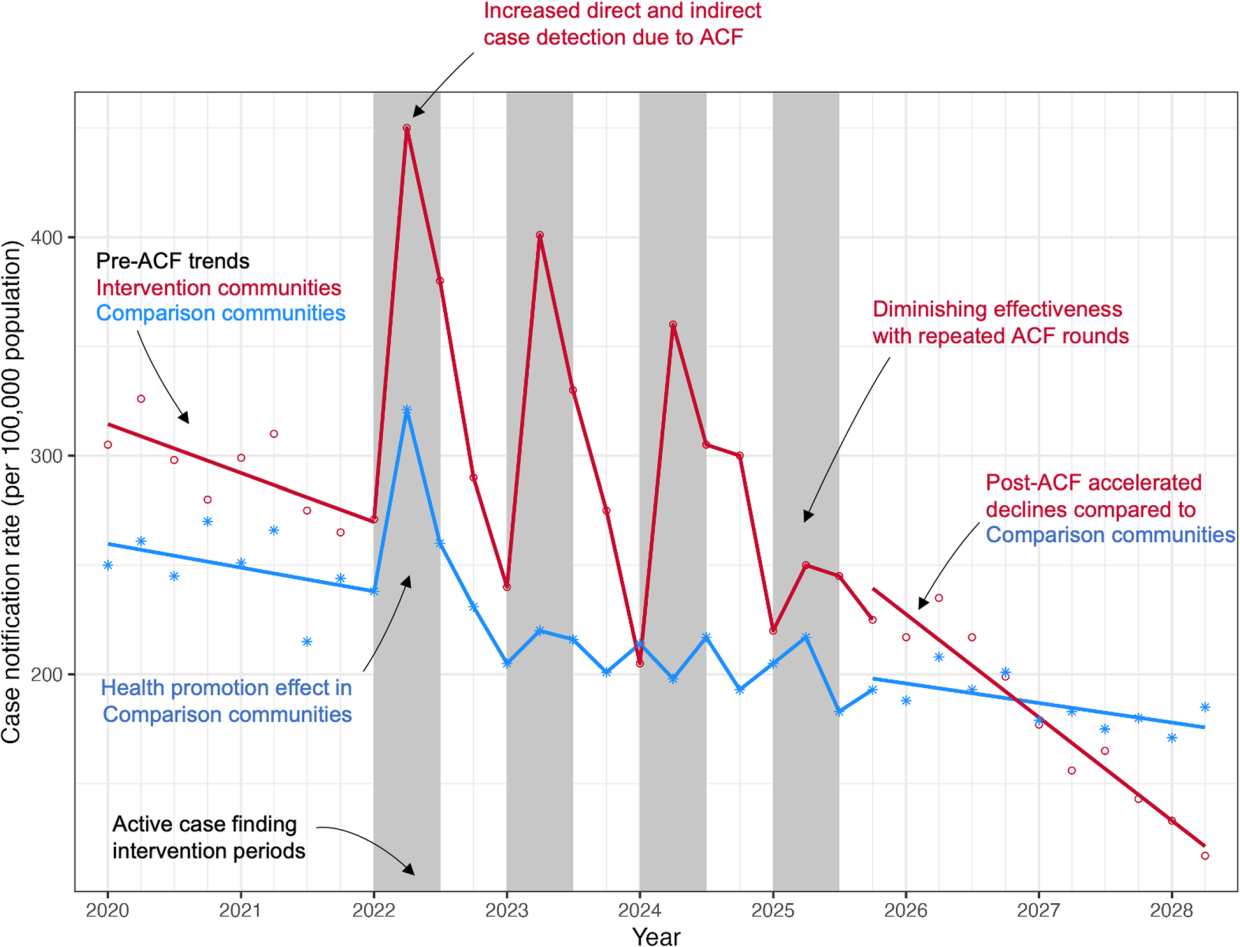
Interpretation of trends in tuberculosis case notification rates following implementation of active case-finding programmes. Note: The figure is illustrative, but based on data from Blantyre, Malawi, analysed by Burke et al. [38]. ACF active case finding

Alternative outcome measures include a comparison of trends in TB case notification rates, but accurate interpretation can be difficult (Fig. 2). Analysis of case notification rates requires high-quality case ascertainment and stable case definitions over time, which can be challenging when only monthly or quarterly aggregated counts of notifications are recorded by District and National TB Programmes. WHO efforts to improve individual-level recording and reporting of TB notifications using standardised electronic forms are welcomed, and we have previously demonstrated the benefits of a low-cost and easy-to-implement surveillance system at the city-level in Blantyre, Malawi [76]. Where a detailed assessment of TB registers shows high-quality capture of TB notifications, we recommend that several years of pre-ACF trends are plotted and interrogated, stratified by key case characteristics including age, sex, HIV status, and TB microbiological status, as these will give an indication of the reliability of pre-ACF case ascertainment. Seasonal fluctuations in trends are to be anticipated [77]. Comparison of case notification rates additionally requires up-to-date delineation of community boundaries and population counts (with age-sex pyramids) to allow accurate projection of population denominator growth for the periods before, during, and after ACF implementation. Finally, a reliable means of allocating people notified with TB to a community boundary is required, taking care to minimise bias. In Malawi, where municipal address systems are lacking, we developed an innovative electronic geolocation application using high-resolution satellite mapping and community-led annotation of landmarks [76, 78]. Regular quality assurance of notification data and geolocation to communities is strongly recommended.

Alternative approaches that could be used to evaluate the epidemiological impact of community-based ACF programmes include measurement of Mtb immunoreactivity in sentinel population groups [75]. Although at the individual level, immunological evidence of exposure to Mtb has at best only moderate predictive precision for identifying individuals who will progress to development of TB [79], at the population level, distributions of positive tuberculin skin tests, IGRAs, or newer tests can give an “epidemiological signal”. Particularly for younger children—who by definition have been recently exposed—this can provide supportive evidence of the effect of ACF programmes on TB transmission. However, Mtb immunoreactivity surveys are logistically challenging, requiring detailed management plans to support children with positive results, and it is not clear whether they are acceptable to communities and families.

There has been some interest in investigating whether genomic epidemiology to investigate putative transmission chains from whole genome sequencing of *Mtb* isolates could provide a novel approach to investigating the impact of community-based ACF on TB transmission [80]. However, despite the increasing availability of sequencing in high TB-burden countries and advanced modelling approaches to investigate transmission chains, major challenges remain. These include the often long incubation period of TB, meaning that prolonged periods of population follow-up may be required to identify transmission effects through analysis of genomically linked cases; continued suboptimal case detection and culture confirmation of cases, even where ACF programmes are ongoing, meaning that many transmission events may be missed; and the current lack of ability to undertake sequencing directly from sputum samples without culture, hugely increasing costs and laboratory infrastructure required. Our position therefore is that, in the high-burden TB settings where ACF is likely to provide epidemiological benefit, the added costs and logistical difficulties of whole genome sequencing analysis, and the substantial issues with potential bias in case and sequencing ascertainment, mean that currently this approach probably provides little additional actionable insights. In settings where TB epidemics are becoming substantially more concentrated, and in outbreak investigations, whole genome sequencing likely has a greater role to play in directing public health response [81].

Given the paucity of evidence identified for the individual-level impacts of ACF on participants and communities [41, 42], we strongly recommend that investigation of these outcomes is embedded within the implementation and evaluation of programmes. This could be efficiently done through surveys (perhaps through cross-sectional surveys) with random samples of participants undergoing screening, nested case-control studies to compare knowledge, attitudes and understanding, and qualitative and focus group discussion work to investigate acceptability.

We recognise that, although more randomised trials of community-based ACF programmes—including existing and new tools—are required, not all programmes have the resources to implement and evaluate trials. Nevertheless, where ACF is being undertaken, a rigorous monitoring and evaluation strategy is still required. Where randomisation of communities is not possible and interventions are allocated to communities by investigators or public health programmes, we recommend comparable communities are selected as a control group, and that statistical approaches to attempt to account for selection bias are used, although recognising that these are unlikely to be able to completely overcome differences. Studies that only evaluate the yield of TB diagnosis or change in case notification rates from before to during ACF introduction will add little to existing knowledge about ACF effectiveness and may give a falsely optimistic picture of programme impact [22]. With prolonged (several years) follow-up beyond the ACF period, analysis of time trends in case notification rates may provide some additional information, particularly where the post-ACF period trends show an accelerated decline compared to the pre-ACF period (Fig. 2), although it remains challenging to discount the effect of other temporal determinants.

## What ACF is not

The above discussion has made it clear that we are strongly supportive of the role of ACF in making progress toward the goals of TB elimination when it is offered in the right places and at the right times. However, all the above evidence and our own experience make it clear that TB ACF is neither a one-size-fits-all intervention nor a panacea.

We have reviewed the potential community-level harms of ACF, largely to do with opportunity cost, value for money, and use of resources. There is insufficient evidence about the potential for individual harms of ACF, particularly if ACF is not well targeted to a group of people with a high pre-test probability of TB disease and particularly as ACF programmes attempt to detect more people with subclinical TB disease. Furthermore, TB ACF cannot and should not be a substitute for equitable access to responsive, affordable, and accessible primary care services for all, including TB [82, 83]. Where people who perceive themselves to be unwell are unable to access primary care routinely and receive a TB diagnosis there, TB services in primary care should be funded and strengthened in preference to spending money on temporary ACF services and campaigns.

## Conclusions

Community-based active case finding for TB is a potentially powerful tool in our armament to accelerate efforts to eliminate TB as a public health problem. However, where TB epidemics are beginning to concentrate, particularly within highly marginalised groups, decisions to implement an ACF programme should be carefully considered, based on a thorough public health needs assessment, and include a comprehensive monitoring and evaluation plan, with nested research to investigate major outstanding knowledge gaps. People and communities affected by TB should remain at the forefront of our consideration and decisions about whether and how to offer TB screening through an ACF programme should involve individuals and communities in decision-making, and carefully consider potential trade-offs between population and individual benefits and harms.

## Data Availability

Not applicable.
