# Author Correction: Community-based active-case finding for tuberculosis: navigating a complex minefield

**DOI:** 10.1186/s44263-024-00048-3

**Published:** 2024-02-16

**Authors:** Peter MacPherson, Kwame Shanaube, Mphatso D. Phiri, Hannah M. Rickman, Katherine C. Horton, Helena R. A. Feasey, Elizabeth L. Corbett, Rachael M. Burke, Molebogeng X. Rangaka

**Affiliations:** 1 School of Health and Wellbeing, University of Glasgow, Glasgow, UK; 2 Clinical Research Department, London School of Hygiene & Tropical Medicine, London, UK; 3 ZAMBART, University of Zambia, Lusaka, Zambia; 4 Malawi-Liverpool-Wellcome Programme, Blantyre, Malawi; 5 Department of Clinical Sciences, Liverpool School of Tropical Medicine, Liverpool, UK; 6 Department of Infectious Disease Epidemiology, London School of Hygiene & Tropical Medicine, London, UK; 7 CIDRI-Africa, University of Cape Town, Cape Town, South Africa; 8 MRC Clinical Trials Unit, University College London, London, UK

**Author Correction: BMC Global Public Health 2, 9 (2024)**

**https://doi.org/10.1186/s44263-024-00042-9**

Following publication of the original article it was reported that Kwame Shanaube’s name was misspelt ‘Shaunabe’. The original article has been updated.
